# Risk Stratification by Coronary Perfusion Pressure in Left Ventricular Systolic Dysfunction Patients Undergoing Revascularization: A Propensity Score Matching Analysis

**DOI:** 10.3389/fcvm.2022.860346

**Published:** 2022-04-14

**Authors:** Ming-Jer Hsieh, Chun-Chi Chen, Dong-Yi Chen, Cheng-Hung Lee, Ming-Yun Ho, Jih-Kai Yeh, Yu-Chang Huang, Yu-Ying Lu, Chieh-Yu Chang, Chao-Yung Wang, Shang-Hung Chang, I-Chang Hsieh

**Affiliations:** ^1^Division of Cardiology, Department of Internal Medicine, Chang Gung Memorial Hospital, Taoyuan, Taiwan; ^2^College of Medicine, Chang Gung University, Taoyuan, Taiwan; ^3^Center for Big Data Analytics and Statistics, Department of Medical Research and Development, Chang Gung Memorial Hospital, Taoyuan, Taiwan

**Keywords:** coronary perfusion pressure, complete revascularization, reasonable incomplete revascularization, residual SYNTAX score, left ventricular systolic dysfunction

## Abstract

**Background:**

Coronary perfusion pressure (CPP) and coronary artery stenosis are responsible for myocardial perfusion. However, how CPP-related survival outcome affects revascularization is unclear.

**Objective:**

The aim of this study is to investigate the prognostic role of CPP in patients with left ventricular systolic dysfunction (LVSD) undergoing percutaneous coronary intervention (PCI) with complete revascularization (CR) or reasonable incomplete revascularization (RIR).

**Methods:**

We retrospectively screened 6,076 consecutive patients in a registry. The residual synergy between percutaneous coronary intervention with Taxus and cardiac surgery (SYNTAX) score (rSS) was used to define CR (rSS = 0) and RIR (0<rSS≤8). Propensity score matching was performed to reduce bias between RIR and CR. The primary endpoint was all-cause mortality.

**Results:**

In total, 816 patients with LVSD who underwent CR or RIR were enrolled. After a mean follow-up of 4.6 years, 134 patients died. Both CPP and RIR independently predicted mortality in the total population. After 1:1 matching, 175 pairs of RIR and CR were found in patients with CPP > 42 mmHg. Moreover, 101 pairs of RIR and CR were present in patients with CPP ≤ 42 mmHg. In patients with CPP > 42 mmHg, RIR was not significantly different from CR in long-term mortality [hazard ratio (HR) 1.20; 95% confidence interval (CI):0.70–2.07; *p* = 0.513]; However, in patients with CPP≤42 mmHg, RIR had a significantly higher mortality risk than CR (HR 2.39; 95% CI: 1.27–4.50; *p* = 0.007).

**Conclusions:**

The CPP had a risk stratification role in selecting different revascularization strategies in patients with LVSD. When patients with LVSD had CPP > 42 mmHg, RIR was equivalent to CR in survival. However, when patients with LVSD had CPP ≤ 42 mmHg, RIR had a significantly higher mortality risk than CR.

## Introduction

Coronary perfusion pressure (CPP) is the pressure gradient that drives forward coronary flow for myocardial perfusion (1). Coronary blood largely supplies the myocardium during the diastolic phase of the cardiac cycle (2). In the systolic phase, blood flow can follow the systolic blood pressure (SBP) into the epicardial coronary artery. However, the blood flow supply is obstructed due to myocardium contraction. Conversely, when the myocardium relaxes in the diastolic phase, it experiences pressure from both the aorta and left ventricle, and the coronary flow follows the pressure gradient between the aortic diastolic blood pressure (DBP) and left ventricular end-diastolic pressure (LVEDP) to provide myocardium blood supply. Therefore, CPP is defined as the pressure difference between DBP and LVEDP (3). Both DBP and LVEDP can be used as predictors of clinical outcomes in patients with cardiovascular diseases. Low DBP has been associated with subclinical myocardial ischemia and other unfavourable outcomes (4–6). The LVEDP in patients with left ventricular failure not only has an impact on long-term outcomes, but can also be a surrogate of an unloading mechanical device to increase coronary flow (7–9). However, despite being a composite of DBP and LVEDP, the association between CPP and clinical events in patients with heart diseases remains poorly explored.

In addition to CPP, stenosis of the coronary artery is another mechanical factor associated with myocardial perfusion. In the presence of coronary stenotic lesions, a post-stenotic pressure drop will result in flow-limited myocardial hypoperfusion. Regardless of whether coronary bypass surgery or percutaneous coronary intervention (PCI) is applied, achievement of complete revascularisation (CR) is the primary treatment goal, especially for patients with heart failure or left ventricular systolic dysfunction (LVSD). However, limited by patients' comorbidities, anatomical factors, and technical or procedural considerations, CR is only achieved in less than two-thirds of patients either by PCI or coronary bypass surgery in real-world practise (10–12). Recent studies demonstrate that reasonable incomplete revascularization (RIR) is determined by a reasonable assessment method to decide whether a lesion should be treated, such as fractional flow reserve or residual synergy between percutaneous coronary intervention with Taxus and cardiac surgery (SYNTAX) score (rSS) calculation, is equivalent to CR in terms of long-term outcomes (13, 14). The rSS is a systemic angiographic score that can objectively quantify the reasonableness of RIR. It most generally defines a post revascularization score of 0<rSS≤8 as RIR (15).

Considering that both CPP and residual stenosis of coronary arteries after revascularization are responsible for myocardial perfusion, this study retrospectively analysed a real-world registry data and investigated whether CPP had a risk stratification role in patients with LVSD undergoing PCI with either an angiographic CR or RIR strategy.

## Materials and Methods

### Database and Study Population

From January 2003 to December 2017, 6,076 consecutive patients with ischemic heart disease, who underwent PCI and were registered in the Cardiovascular Atherosclerosis and Percutaneous Transluminal Interventions (CAPTAIN) registry, were screened in this study. This registry is a physician-initiated, single-centre, and long-term follow-up registry that includes consecutive patients undergoing elective or emergent PCI. After the index PCI procedure and acquisition of informed consent for data collection, the patient's clinical and procedural data were prospectively entered into a database. Medical records on clinical status, medical management, and occurrence of any adverse events were also obtained. The patients were clinically followed up through outpatient visits or telephonic contact. The Chang Gung Medical Foundation Institutional Review Board approved the protocol of data collection in this study (Approval No. 202101400B0).

**Figure 1:**
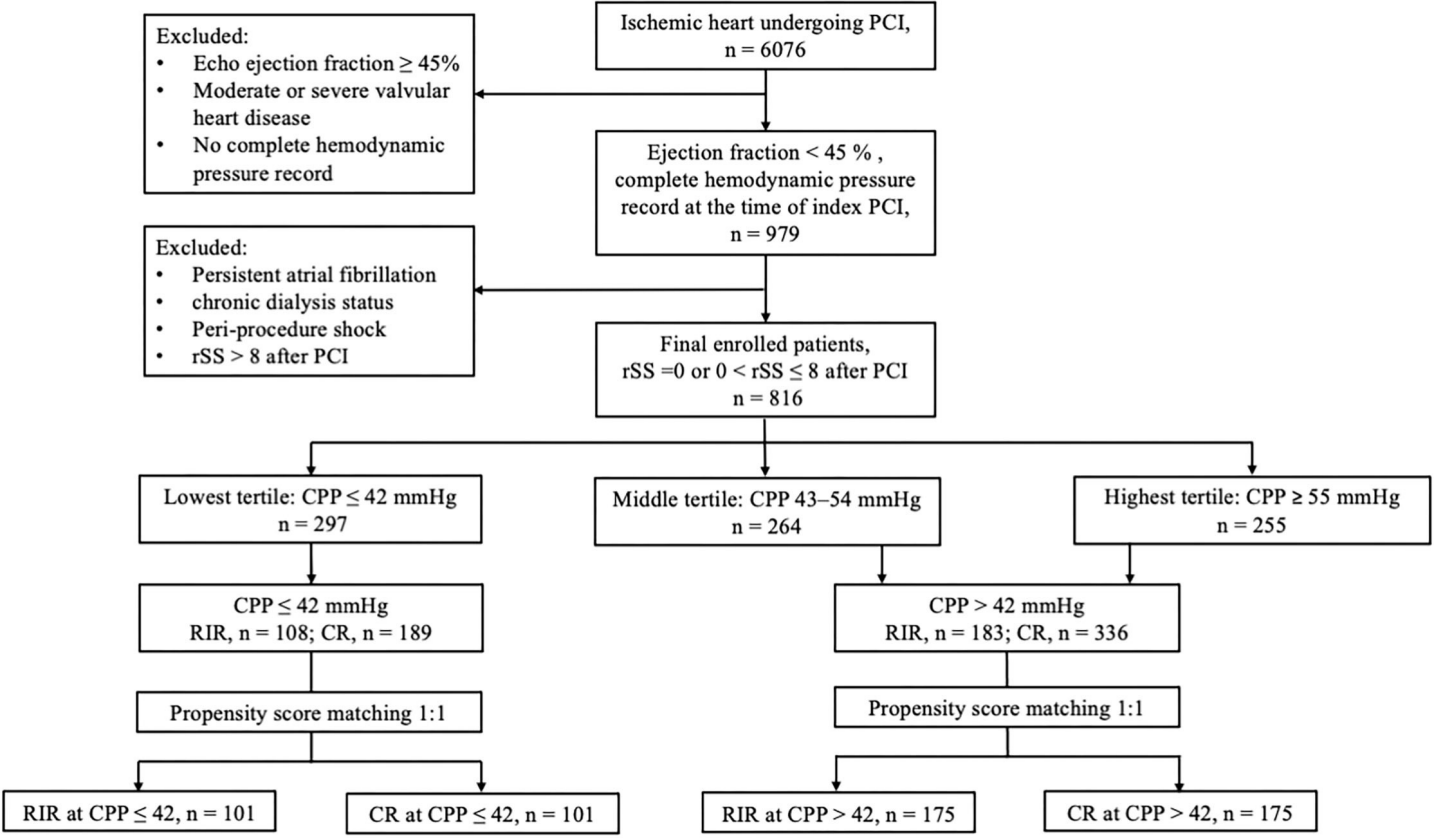
Flowchart of patient enrolment. CPP, coronary perfusion pressure; CR, complete revascularisation; RIR, reasonable incomplete revascularisation; rSS, residual SYNTAX score.

The flowchart of patient enrolment is shown in Figure 1. The following were the inclusion criteria: 1) left ventricular ejection fraction (LVEF) <45% by echocardiography; 2) a complete haemodynamic pressure record before index PCI; and 3) post-PCI rSS ≤ 8. The exclusion criteria were patients with LVEF ≥45%, with moderate-to-severe valvular heart disease, and without complete haemodynamic pressure records at the index PCI. Patients with rSS>8 and conditions that could possibly interfere with the haemodynamic pressure recordings, such as persistent atrial fibrillation, end-stage kidney disease on chronic dialysis status, and shock status peri-index PCI procedure, were also excluded. The primary outcome of this study was all-cause mortality after the index PCI. All patients were followed for 5 years or until 31 December 2019.

### PCI Procedure, Definition, and Haemodynamic Pressure Recording

The methods of PCI procedure and contents of haemodynamic pressure recordings mainly depended on the patient's clinical condition and the physician's decision at the time of index PCI. Angiographic CR and RIR were defined based on rSS after PCI. Angiographic CR was defined as rSS = 0, i.e., there was no stenosis in ≥50% in a segment of at least 2.25 mm diameter after the index PCI. Clinically, RIR (0<rSS≤8) is defined as a treating culprit or ischaemic vessels according to the findings of electrocardiography, echocardiography, or stress tests sparing any coronary artery with ≥50% stenosis that is not a culprit coronary artery, asymptomatic side branch, or a small vessel. Patients with an rSS > 8 were not considered to have RIR and were excluded from our analysis. In general, cardiac haemodynamic pressure recordings were performed in patients with LVSD before the index PCI. Briefly, a 5F or 6F pigtail catheter was inserted *via* the radial artery or common femoral artery through the aortic valve and into the left ventricle, in which LVEDP was recorded. The pigtail catheter was then pulled back from the left ventricle to the aorta, and SBP and DBP were recorded at the ascending aorta. All haemodynamic pressures were recorded by averaging at least three consecutive values.

### Statistical Analysis

All statistical data were analysed using Statistical Package for the Social Sciences (SPSS) version 23. Continuous data are presented as the mean ± standard deviation (SD), and categorical data are presented as numbers and percentages. The *t*-test or Wilcoxon rank-sum test was used for comparisons between groups of continuous data and the chi-square test for categorical data. Cox-regression analysis was performed to identify independent predictors of long-term mortality and relative risk between subgroups after adjusting unbalanced underlying variables. The optimal CPP value to predict mortality was calculated by using Youden‘s index in the receiver operating characteristic curve.

One-to-one propensity score-matched analysis was performed to overcome selection bias for PCI treatment (RIR or CR) and any other related potential covariant bias. Pre-PCI treatment variables including age, gender, diabetes mellitus, smoking, hyperlipidaemia, previous stroke, chronic kidney disease (CKD) stage, New York Heart Association Functional Class (NYHA Fc), LVEF, heart rate, and the presence of calcified and chronic total occlusion lesion were included in binary logistic regression to estimate the probability. The match tolerance was initially set as a width of 0.25 multiplied by the SD of the propensity score distribution. Survival curves were estimated using the Kaplan-Meier method, and the log-rank test was used to examine between-group differences in survival for categorical variables. A *p* < 0.05 was considered to indicate a significant difference.

## Results

### Predictors of Long-Term Mortality

After a mean follow-up period of 4.6 ± 1.2 years, 134 patients (16.4%) died in the total population. After adjusting for age (per 10 years), gender, hypertension, diabetes mellitus, hyperlipidaemia, smoking, CKD stage ≥3, prior stroke, calcified lesion, NYHA Fc ≥3, LVEF <35%, chronic total occlusion lesion, heart rate >70 beat/min, CPP (continuous value, per mmHg increase), beta-blocker therapy, angiotensin-converting enzyme inhibitor (ACEi)/angiotensin receptor blocker (ARB) therapy, and RIR (vs. CR) in multivariable Cox-regression analysis, the independent predictors of long-term mortality were CPP (continuous value, per mmHg increase, HR 0.97; 95% CI:0.95–0.00; *p* = 0.001) and RIR (HR 2.08; 95% CI: 1.27–3.39; *p* = 0.003).

### Patient Characteristics According to CPP Tertile

To investigate the role of CPP in survival, we initially classified the patients with LVSD according to the tertile of CPP level (highest tertile, ≥55 mmHg; intermediate tertile, 43–54 mmHg; and lowest tertile, ≤42 mmHg). Table 1 shows comparisons between these three tertiles in clinical characteristics. In general, patients with lower CPP were older and had less hypertension but more NYHA Fc≥3 and CKD than patients with higher CPP.

**Table 1:** Baseline characteristics of patients with left ventricular systolic dysfunction (LVSD) according to the tertile of coronary perfusion pressure.

**CPP, (range, mmHg)** **medium; mean ±standard deviation, mmHg**	**Lowest tertile, (≤42)** **36; 34.4 ± 6.9**	**Intermediate tertile, (43–54)** **49; 48.6 ±3.4**	**Highest tertile, (≥55)** **63; 65.3 ± 9.9**	***p*** **-value**
Patient number, *n*	297	264	255	
Age, years	65.6 ± 11.7	62.0 ± 11.7	61.8 ± 11.5	<0.001
Female gender, *n* (%)	60 (20.2)	53 (20.1)	48 (18.8)	0.908
Diabetes mellitus, *n* (%)	122 (41.1)	115 (43.6)	87 (34.1)	0.074
Hypertension, *n* (%)	139 (46.8)	149 (56.4)	155 (60.8)	0.003
Hyperlipidemia, *n* (%)	139 (46.8)	148 (56.1)	123 (48.2)	0.067
Smoking, *n* (%)	133 (44.8)	111 (42.0)	112 (43.9)	0.803
CKD ≥3, *n* (%)	59 (19.9)	45 (17.0)	21 (8.2)	<0.001
Previous stroke, *n* (%)	14 (4.7)	18 (6.8)	9 (3.5)	0.219
NYHA Fc ≥3, *n* (%)	73 (24.6)	63 (23.9)	40 (15.7)	0.022
LVEF, %	34.6 ± 7.8	34.9 ± 8.0	35.4 ± 7.8	0.514
LVEF <35%, *n* (%)	139 (46.8)	117 (44.3)	106 (41.6)	0.467
Calcified lesions, *n* (%)	74 (24.9)	62 (23.5)	54 (21.2)	0.582
Chronic total occlusion, *n* (%)	27 (9.1)	23 (8.3)	36 (14.1)	0.089
SBP, mmHg	129.8 ± 24.9	138.3 ± 24.9	150.4 ± 23.6	<0.001
DBP, mmHg	63.8 ± 10.0	73.3 ± 8.2	85.9 ± 12.2	<0.001
Heart rate, beat/min	72.3 ± 12.0	75.1 ± 14.6	75.8 ± 13.9	0.005
LVEDP, mmHg	29.4 ± 8.3	24.7 ± 8.0	20.5 ± 9.1	<0.001
rSS, mean	2.2 ± 3.2	2.1 ± 3.1	2.2 ± 3.1	0.862
RIR (rSS: 5 to 8), *n* (%)	84 (28.3)	68 (25.8)	65 (25.5)	0.709
RIR (rSS: 1 to 4), *n* (%)	24 (8.1)	24 (9.1)	25 (9.8)	0.775
CR (rSS = 0), *n* (%)	189 (63.6)	172 (65.2)	164 (64.3)	0.932
ACEi or ARB, *n* (%)	242 (81.5)	219 (83.0)	219 (85.9)	0.377
Beta-blocker, *n* (%)	262 (88.2)	235 (89.0)	233 (91.4)	0.465

*ACEi, angiotensin-converting enzyme inhibitor; ARB, angiotensin receptor blocker; CKD, chronic kidney disease; CPP, coronary perfusion pressure; CR, complete revascularization; DBP, diastolic blood pressure; LVEDP, left ventricular end-diastolic pressure; LVEF, left ventricular ejection fraction; NYHA Fc, New York Heart Association functional class; RIR, reasonable incomplete revascularization; rSS, residual SYNTAX score; SBP, systolic blood pressure*.

### Incidence Rates and Risks of Long-Term Mortality in the CPP Tertiles

The incidence rates and risks of long-term mortality in the CPP tertiles are shown in Table 2. The mortality rates in the highest, intermediate, and lowest tertiles were 13.3%, 13.3%, and 21.9%, and the incidence rates were 28.7, 28.6, and 49.7 per 1,000 person-years, respectively. After adjusting with baseline characteristics, the risk of mortality in the intermediate tertile was not significantly different from that in the highest tertile (crude hazard ratio = 1; 95% CI:0.62–1.60; *p* = 0.998; adjusted hazard ratio = 0.94; 95% CI:0.58–1.52; *p* = 0.798). However, the lowest tertile had a significantly higher risk of mortality than the highest tertile (crude hazard ratio = 1.73; 95% CI: 1.14–2.62; *p* = 0.01; adjusted hazard ratio = 1.63; 95% CI: 1.06–2.49; *p* = 0.026). The receiver-operating characteristic curve demonstrated that CPP cut-off at 42 mmHg had the best prognostic accuracy for predicting mortality (area under the curve 0.64, 95% CI:0.56–0.72, *p* < 0.001).

**Table 2:** Incidence and risk of mortality according to the tertile of coronary perfusion pressure (CPP).

**Tertile of CPP (mmHg)**	**Patient number, ***n*****	**Events, ***n*** (%)**	**Incidence per 1,000 person-years**	**Unadjusted hazard ratio (95% CI)**	***p*** **-value**	**Adjusted hazard ratio (95% CI)**	***p*** **-value**
Highest (≥55)	255	34 (13.3)	28.7	1.00 [Reference]	–	1.00 [Reference]	–
Intermediate (43–54)	264	35 (13.3)	28.6	1.00 (0.62–1.60)	0.998	0.94 (0.58–1.52)	0.798
Lowest ( ≤ 42)	297	65 (21.9)	49.7	1.73 (1.14–2.62)	0.010	1.63 (1.06–2.49)	0.026

*CI, confidence interval; CPP, coronary perfusion pressure*.

*Adjusted for clinical variables including age (per 10 years), gender, hypertension, diabetes mellitus, hyperlipidemia, smoking, previous stroke, chronic kidney disease, NYHA Fc ≥3, left ventricular ejection fraction <35%, calcified lesions, systolic blood pressure, heart rate, complete revascularization, use of angiotensin-converting enzyme inhibitor or angiotensin receptor blocker, and beta-blockers in a Cox proportional regression model*.

### Baseline Characteristics of the Four Subgroups in the Total and Matched Populations

Due to significant differences between low and high CPP in baseline characteristics, patients were divided into 2 parts according to CPP (>42 or ≤ 42 mmHg). Then, RIR was compared to CR within each part (>42 or ≤ 42 mmHg), respectively. Finally, all patients were further divided into four subgroups: CR at CPP>42, RIR at CPP>42, CR at CPP ≤ 42, and RIR at CPP ≤ 42 to explore the interaction between CPP and revascularization status. Supplementary Table 1 shows the comparisons of baseline characteristics between the four subgroups in the total population.

To minimalize the confounding effect of baseline characteristics, propensity score matching was performed in patients with CPP>42 mmHg and ≤ 42 mmHg, respectively. After 1:1 matching, 175 pairs of RIR vs. CR in patients with CPP>42 mmHg were found. Moreover, 101 pairs of RIR vs. CR in patients with CPP ≤ 42 mmHg were present. Table 3 shows the comparisons of baseline characteristics between subgroups in the matched population. In comparisons of RIR and CR, either in patients with CPP>42 or ≤ 42 mmHg, none of the variables significantly differed.

**Table 3:** Baseline characteristics of matched population according to CPP and revascularization status.

	**CPP≤42 mmHg**				**CPP>42 mmHg**				***p*** **-value, CPP ≤42 vs. >42**
	**Total** **(*n* = 202)**	**RIR** **(*n* = 101)**	**CR** **(*n* = 101)**	***p*****-value,** **RIR vs. CR**	**Total** **(*n* = 350)**	**RIR** **(*n* = 175)**	**CR** **(*n* = 175)**	***p*****-value,** **RIR vs. CR**	
Age, years old	67.2 ± 11.6	67.6 ± 10.7	66.9 ± 12.6	0.639	62.2 ± 11.5	62.0 ± 11.3	62.4 ± 11.7	0.773	<0.001
Female gender, *n* (%)	42 (20.8)	23 (22.8)	19 (18.8)	0.603	79 (22.6)	39 (22.3)	40 (22.9)	1.000	0.670
Diabetes mellitus, *n* (%)	96 (47.5)	47 (46.5)	49 (48.5)	0.888	162 (46.3)	83 (47.4)	79 (45.1)	0.748	0.791
Hypertension, *n* (%)	107 (53.0)	54 (53.5)	53 (52.5)	1.000	240 (68.6)	119 (68.0)	121 (69.1)	0.908	<0.001
Hyperlipidaemia, *n* (%)	96 (47.5)	51 (50.5)	45 (44.6)	0.481	175 (50.0)	97 (55.4)	78 (44.6)	0.054	0.597
Smoking, *n* (%)	89 (44.1)	45 (44.6)	44 (43.6)	1.000	133 (38.0)	75 (42.9)	58 (33.1)	0.078	0.177
CKD stage ≥3, *n* (%)	42 (20.8)	20 (19.8)	22 (21.8)	0.863	55 (15.7)	28 (16.0)	27 (15.4)	1.000	0.133
Previous stroke, *n* (%)	12 (5.9)	7 (6.9)	5 (5.0)	0.767	16 (4.6)	9 (5.1)	7 (4.0)	0.799	0.547
NYHA Fc ≥3, *n* (%)	56 (27.7)	30 (29.7)	26 (25.7)	0.637	76 (21.7)	42 (24.0)	34 (19.4)	0.364	0.121
LVEF, %	34.0 ± 8.0	34.0 ± 8.2	34.0 ± 7.9	0.986	34.4 ± 8.3	34.6 ± 8.4	34.3 ± 8.3	0.798	0.518
LVEF <35, *n* (%)	97 (48.0)	50 (49.5)	47 (46.5)	0.778	164 (46.9)	81 (46.3)	83 (47.4)	0.915	0.860
Calcified lesion, *n* (%)	55 (27.2)	28 (27.7)	27 (26.7)	1.000	83 (23.7)	45 (25.7)	38 (21.7)	0.451	0.361
Chronic total occlusion, *n* (%)	18 (8.9)	7 (6.9)	11 (10.9)	0.460	40 (11.4)	17 (9.7)	23 (13.1)	0.401	0.390
ACEi or ARB, *n* (%)	166 (82.2)	84 (83.2)	82 (81.2)	0.854	295 (84.3)	145 (82.9)	150 (85.7)	0.557	0.552
Beta-blocker, *n* (%)	178 (88.1)	88 (87.1)	90 (89.1)	0.828	316 (90.3)	159 (90.9)	157 (89.7)	0.857	0.472
SBP, mmHg	131.2 ± 24.8	132.3 ± 26.4	130.1 ± 23.3	0.531	144.9 ± 25.1	145.6 ± 24.7	144.3 ± 25.5	0.639	<0.001
DBP, mmHg	63.3 ± 10.0	62.5 ± 9.9	64.2 ± 10.1	0.217	79.1 ± 11.5	79.1 ± 11.0	79.1 ± 12.0	0.974	<0.001
Heart rate, beat/min	72.3 ± 12.1	73.0 ± 12.3	71.6 ± 11.9	0.410	75.9 ± 14.1	75.4 ± 13.8	76.4 ± 14.4	0.492	0.003
LVEDP, mmHg	29.4 ± 8.6	28.4 ± 9.1	30.5 ± 8.0	0.086	22.4 ± 9.1	21.9 ± 9.6	22.8 ± 8.5	0.359	<0.001
CPP, mmHg	33.9 ± 8.0	34.1 ± 6.1	33.7 ± 8.0	0.744	56.8 ± 11.0	57.2 ± 10.9	56.3 ± 11.2	0.431	<0.001

*ACEi, angiotensin-converting enzyme inhibitor; ARB, angiotensin receptor blocker; CKD, chronic kidney disease; CPP, coronary perfusion pressure; DBP, diastolic blood pressure; LVEDP, left ventricular end-diastolic pressure; LVEF, left ventricular ejection fraction; NYHA Fc, New York Heart Association functional class; SBP, systolic blood pressure*.

### Incidence Rates and Relative Risks of Long-Term Mortality in Total and Matched Population

Table 4 shows the incidence rate and relative mortality risk between subgroups. In the total population, the incidence rates of mortality in CR at CPP>42, RIR at CPP>42, CR at CPP ≤ 42, and RIR at CPP ≤ 42 were 11.3%, 16.9%, 17.5%, and 29.6%, respectively. In the propensity-matched population, the incidence rates of mortality in CR at CPP>42, RIR at CPP>42, CR at CPP ≤ 42, and RIR at CPP ≤ 42 were 13.7%, 16.0%, 13.9%, and 29.7%, respectively. In the analysis of matched population showed in patients with CPP>42 mmHg, the mortality risk between RIR and CR did not significantly differ (HR 1.20; 95% CI: 0.70–2.07; *p* = 0.513), but in patients with CPP ≤ 42 mmHg, RIR was associated with a significantly higher mortality risk than CR (HR 2.39; 95% CI: 1.27–4.50; *p* = 0.007).

**Table 4:** Incidence and risk of long-term mortality between subgroups.

**CPP level, mmHg**	**PCI strategy**	**Total population**			**Matched population**						
		**Total number, *n***	**Event number, *n***	**Incidence, %**	**Total numbers, *n***	**Event number, *n***	**Incidence, %**	**HR (95% CI) within CPP >42 or ≤42 mmHg**	***p*** **-value**	**Adjusted HR (95% CI) between 4 subgroups**	***p*** **-value**
>42	CR	336	38	11.3	175	24	13.7	1.00 [Reference]	–	1.00 [Reference]	–
	RIR	183	31	16.9	175	28	16.0	1.20 (0.70–2.07)	0.513	1.11 (0.65–1.93)	0.695
≤ 42	CR	189	33	17.5	101	14	13.9	1.00 [Reference]	–	0.99 (0.51–1.91)	0.965
	RIR	108	32	29.6	101	30	29.7	2.39 (1.27–4.50)	0.007	2.12 (1.23–3.66)	0.007

*CI, confidence interval; CPP, coronary perfusion pressure; CR, complete revascularisation; HR, hazards ratio; PCI, percutaneous coronary interventions; RIR, reasonable incomplete revascularisation*.

**Figure 2:**
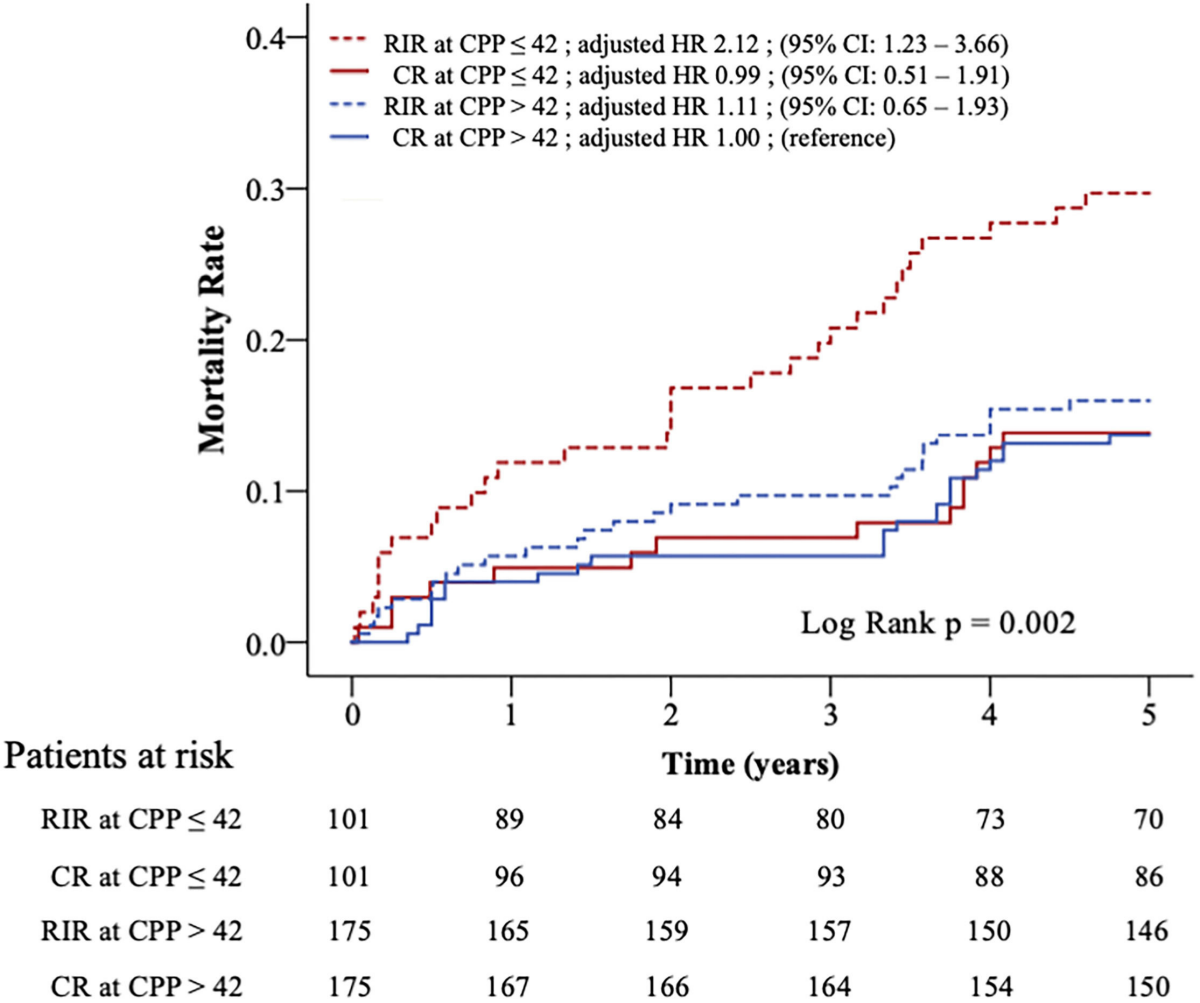
Kaplan–Meier curves and relative risks for all-cause mortality in four subgroups. Patients in propensity-matched population were divided into four subgroups according to CPP (>42 or ≤ 42 mmHg) and revascularisation strategy (RIR or CR). The relative risks of mortality between the four subgroups were adjusted in Cox-proportional hazards analysis. CPP, coronary perfusion pressure; CR, complete revascularisation; RIR, reasonable incomplete revascularisation; * means *p* < 0.05.

Figure 2 shows the Kaplan-Meier survival curves of the four subgroups (log-rank *p* = 0.002). Clinical variables were adjusted in the Cox model for comparing the relative mortality risk between the four subgroups in the matched population (Table 4). The CR at CPP>42, RIR at CPP>42, and CR at CPP ≤ 42 did not significantly differ in mortality risk. However, RIR at CPP ≤ 42 had a significantly higher mortality risk among these four subgroups (adjusted HR 2.12; 95% CI: 1.23–3.66; *p* = 0.007).

## Discussion

There are three main findings in the current study. First, CPP and RIR (0<rSS≤8) were independent predictors of long-term mortality in patients with LVSD. Second, when patients with LVSD had a low CPP ( ≤ 42 mmHg), RIR was worse than CR in long-term survival. Third, RIR could only be comparable to CR in long-term survival when patients had a CPP of >42 mmHg. Therefore, we concluded that CR is crucial for patients with LVSD, whose CPP was ≤ 42 mmHg. The RIR was only equivalent to CR in terms of long-term survival when CPP exceeded 42 mmHg. This is the first study to demonstrate that CPP, measured using an invasive catheter, has a risk stratification role in selecting different revascularization strategies in patients with LVSD.

The CPP is the general presentation of a patient‘s age, comorbidities, and haemodynamic conditions. The interactions between CPP, regulating coronary flow, and heart function are complex and insufficiently understood (16, 17). When the CPP remains between 40 and 120 mmHg, the coronary autoregulation function through microvascular dilation or contraction can modify vascular resistance to maintain a consistent coronary flow (18, 19). If the pressure is below this threshold (about 40 mmHg) and even if the coronary vascular bed seems to be fully dilated, an increase or decrease in coronary flow directly corresponds to a linear increase or decrease in CPP (20, 21). Canty et al. reported that when CPP is <40 mmHg, myocardial wall thickening and segmental shortening will decrease from normal to akinesis (22). Any reduction in resting coronary flow will impact myocardium performance. These results indicate that the myocardium is very sensitive and cannot tolerate resting coronary flow reduction (22, 23). We assumed that CPP will markedly affect the clinical outcomes in patients with LVSD. Therefore, we selected this patient population in this study.

Most previous studies have discussed myocardial perfusion or coronary flow rather than CPP due to intrinsic myocardial autoregulation mechanisms. Johnson et al. investigated 188 patients after 213 revascularizations with either PCI or coronary bypass surgery in a cardiac positron emission tomography database (24). Despite improvements in stress-induced perfusion defects after revascularization, resting myocardial perfusion did not significantly change. They hypothesised that in most patients, autoregulation implies that resting perfusion measurements are of limited value in estimating treatment response. We agree with their hypothesis; however, Johnson's study does not adequately account for patients with extremely low CPP levels. When patients with a low resting CPP, which is below the lower limit of autoregulation threshold, such as those in the lowest tertile (median CPP = 36 mmHg, mean CPP = 34.4 mmHg) of this present study, they are to have a coronary flow far lower than the consistent flow to maintain adequate myocardial perfusion. Even though myocardial perfusion scanning was not performed, patients with LVSD with a low resting CPP should be considered to have insufficient myocardial perfusion and a worse prognosis.

Dr. Böhm et al. demonstrated that lower DBP in patients with stenotic coronary lesions is associated with worse adverse cardiovascular event rates (25). However, after coronary revascularization, the increased risk with low DBP is not observed. Hence, they suggest that DBP potentially involves CPP and myocardial perfusion and is associated with clinical outcomes. However, they do not describe any reperfusion strategy or post-PCI patency of the coronary arteries. The present study emphasises the importance of CR in patients with low CPP. When the CPP is below the threshold to maintain coronary autoregulation function or if the coronary microvascular bed had been maximally dilated, coronary blood flow may begin to decrease when the coronary lesion stenosis exceeds 50% (26). Hence, patients with an extremely low CPP have reduced flow regulation function, and their myocardial perfusion is very sensitive to the patency of coronary arteries. Only performing RIR with 0<rSS≤8 in patients with low CPP may not good enough to improve outcomes. Therefore, CR (rSS = 0) for these patients with low CPP ( ≤ 42 mmHg) is essential.

Pursuing CR in all patients with ischemic heart disease is reasonable and feasible. However, the patient's general condition, comorbidities, or lesion characteristics, such as fragile, heart failure, CKD, acute coronary syndrome presentation, calcified lesions, and chronic total occlusion, may increase the risk of the procedure and limit the benefit and achievement of angiographic CR (27–30). Although the use of mechanical circulation support during non-emergency high-risk PCI can enhance tissue perfusion and achieve higher success rates of angiographic CR, the efficacy of aggressive PCI on improving outcomes is debatable (31, 32). The RIR may have comparable outcomes with angiographic CR in patients undergoing PCI under demanding clinical settings (28, 33). The RIR is indicated in specific clinical situations according to physiological, functional, or anatomic criteria (34). The physiological criteria of RIR include sparing stenotic vessels with fractional flow reserve >0.8, while the functional criteria include sparing stenotic vessels supplying non-viable or small territories of the myocardium. The anatomic criteria include sparing a stenotic lesion that is a non-culprit lesion located at a small vessel or asymptomatic side branch. In general, rSS was an acceptable method to quantify the anatomic definition of RIR. However, the present results challenge this definition. For patients with low CPP ( ≤ 42 mmHg), anatomic rSS criteria (0<rSS≤8) might be insufficient to access the reasonableness of revascularization. Functional or physiological-guided PCI can decrease unnecessary interventions, but previous studies have not considered the impact of low CPP or autoregulation dysfunction on the fractional flow reserve ratio. Further studies are needed to elucidate whether functional or physiological-guided RIR is comparable to angiographic CR in long-term outcomes in patients with low CPP.

### Study Limitations

First, the study used a real-world database and did not perform a randomised comparison. The physicians chose the revascularization strategy according to their own experiences and the patients' conditions. Although we used propensity scores to adjust for all possible clinical factors, hidden bias may still have occurred. Second, we hypothesised that low CPP may result in low coronary flow and myocardial perfusion, contributing to adverse events in patients with LVSD. Unfortunately, we did not directly measure the coronary flow or myocardial perfusion. Finally, given the limited number of patients in this study, future large-scale studies are needed to investigate whether using a lower rSS cut-off point, such as 0<rSS≤4, is suitable to define RIR for patients with low CPP.

## Conclusions

**Figure 3:**
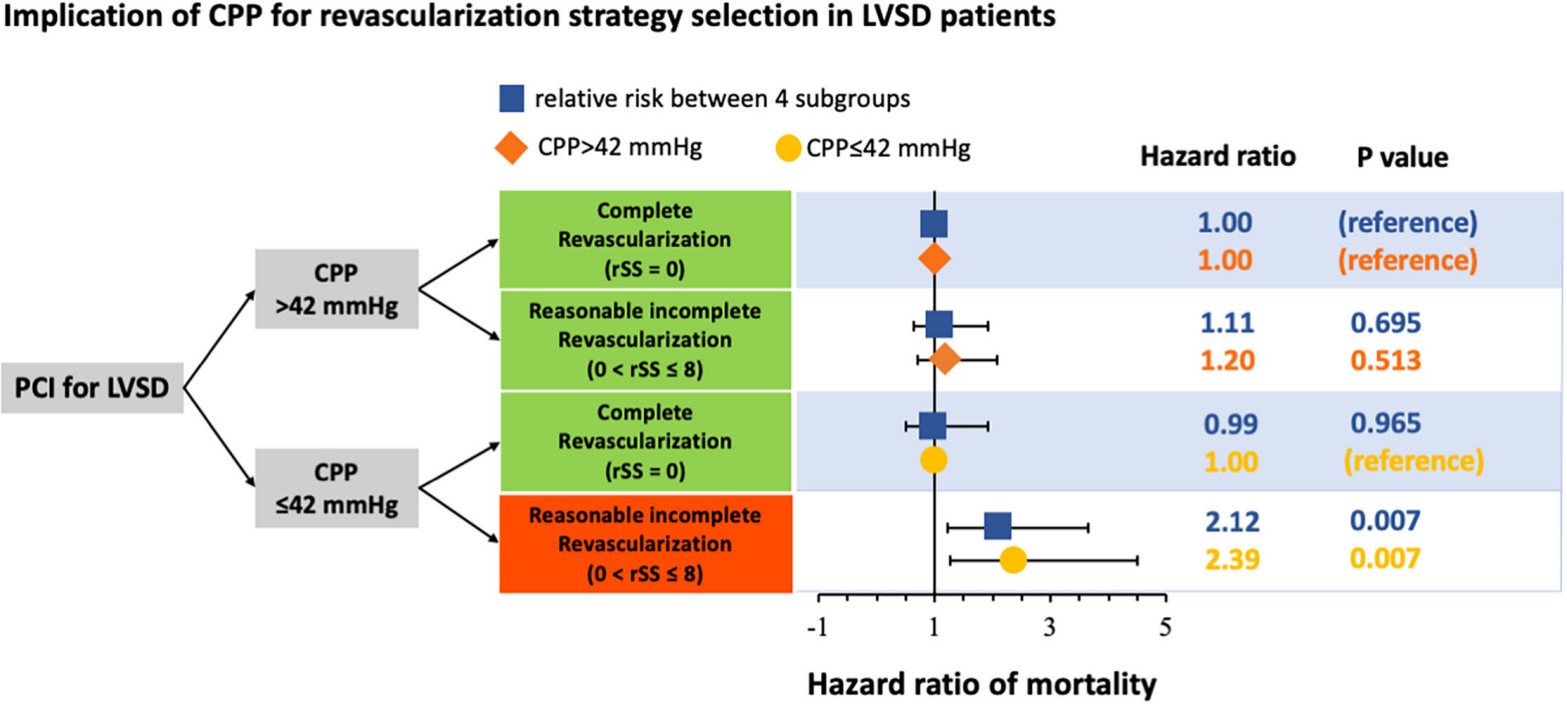
Schematic representation of the implication of coronary perfusion pressure (CPP) for revascularisation strategy selection in patients with left ventricular systolic dysfunction (LVSD) undergoing percutaneous coronary intervention (PCI). CPP, coronary perfusion pressure; LVSD, left ventricular systolic dysfunction; PCI, percutaneous coronary intervention.

Reasonably incomplete revascularization (0<rSS≤8) was known to achieve a comparable outcome and alternative therapy for patients with difficulty achieving CR (rSS = 0) in PCI. This study showed that CPP had a risk stratification role in selecting revascularization strategy in patients with patients with LVSD, as shown in Figure 3. The outcomes of patients with LVSD with CPP > 42 mmHg were consistent with previous studies, showing that RIR was equivalent to CR in long-term mortality. However, when patients with LVSD had CPP ≤ 42 mmHg, RIR was significantly worse than CR in long-term survival. Performing RIR was only inappropriate in patients with LVSD with CPP ≤ 42 mmHg. Additionally, CR should be attempted in this patient subgroup.

## Data Availability Statement

The raw data supporting the conclusions of this article will be made available by the authors, without undue reservation.

## Ethics Statement

The studies involving human participants were reviewed and approved by Chang Gung Medical Foundation Institutional Review Board (Approval No. 202101400B0). Written informed consent was not provided because this study analysed medical records retrospectively.

## Author Contributions

M-JH and I-CH: conceptualisation. M-JH, C-CC, and D-YC: methodology. M-JH, CC-C, D-YC, C-HL, M-YH, J-KY, Y-CH, Y-YL, C-YC, C-YW, S-HC, and I-CH: formal analysis and investigation. M-JH and I-CH: writing review and editing. I-CH: funding acquisition and supervision. All authors contributed to the article and approved the submitted version.

## Funding

This work was supported by grants from Chang Gung Medical Research Program (Grant Numbers CORPG 3C0162).

## Conflict of Interest

The authors declare that the research was conducted in the absence of any commercial or financial relationships that could be construed as a potential conflict of interest.

## Publisher's Note

All claims expressed in this article are solely those of the authors and do not necessarily represent those of their affiliated organizations, or those of the publisher, the editors and the reviewers. Any product that may be evaluated in this article, or claim that may be made by its manufacturer, is not guaranteed or endorsed by the publisher.
