# Supplementary material for: Risk Stratification by Coronary Perfusion Pressure in Left Ventricular Systolic Dysfunction Patients Undergoing Revascularization: A Propensity Score Matching Analysis
Source: Front Cardiovasc Med. 2022 Apr 14;9:860346. doi: 10.3389/fcvm.2022.860346 (PMC9046789; doi:10.3389/fcvm.2022.860346)
Supplement: Supplementary file 1 [file Table_1.DOCX]

**Supplement table S1.** Baseline characteristics of total population according to CPP and revascularization status

|  |  | CPP≤42mmHg | | | |  | CPP>42mmHg | | | |  | p-value,  CPP≤42 vs. >42 |
| --- | --- | --- | --- | --- | --- | --- | --- | --- | --- | --- | --- | --- |
|  |  | Total  (n=297) | RIR  (n=108) | CR  (n =189) | p value,  RIR vs. CR |  | Total  (n=519) | RIR  (n=183) | CR  (n=336) | p value,  RIR vs. CR |  |  |
| Age, years old |  | 65.6±11.8 | 68.0±10.6 | 64.2±12.2 | 0.007 |  | 61.9±11.6 | 61.9±11.2 | 61.9±11.8 | 0.980 |  | <0.001 |
| Female gender, n (%) |  | 60 (20.2) | 23 (21.3) | 37 (19.6) | 0.765 |  | 101 (19.5) | 43 (23.5) | 58 (17.3) | 0.104 |  | 0.855 |
| Diabetes mellitus, n (%) |  | 122 (41.1) | 53 (49.1) | 69 (36.5) | 0.038 |  | 202 (38.9) | 91 (49.7) | 111 (33.0) | <0.001 |  | 0.553 |
| Hypertension, n (%) |  | 139 (46.8) | 60 (55.6) | 79 (41.8) | 0.029 |  | 304 (58.6) | 127 (69.4) | 177 (52.7) | <0.001 |  | 0.001 |
| Hyperlipidaemia, n (%) |  | 135 (45.5) | 52 (48.1) | 83 (43.9) | 0.545 |  | 26.3 (50.7) | 100 (54.6) | 163 (48.5) | 0.199 |  | 0.167 |
| Smoking, n (%) |  | 133 (44.8) | 46 (42.6) | 87 (46.0) | 0.628 |  | 223 (43.0) | 77 (42.1) | 146 (43.5) | 0.781 |  | 0.660 |
| CKD stage ≥3, n (%) |  | 59 (19.9) | 22 (20.4) | 37 (19.6) | 0.881 |  | 66 (12.7) | 36 (19.7) | 30 (8.9) | 0.001 |  | 0.008 |
| Previous stroke, n (%) |  | 14 (4.7) | 7 (6.5) | 7 (3.7) | 0.393 |  | 27 (5.2) | 10 (5.5) | 17 (5.1) | 0.838 |  | 0.868 |
| NYHA Fc ≥3, n (%) |  | 73 (24.6) | 35 (32.4) | 38 (20.1) | 0.025 |  | 103 (19.8) | 43 (23.5) | 60 (17.9) | 0.135 |  | 0.132 |
| LVEF, % |  | 34.6±7.8 | 33.3±8.3 | 35.3±7.4 | 0.033 |  | 35.2±7.9 | 34.6±8.3 | 35.5±7.7 | 0.247 |  | 0.326 |
| LVEF <35, n (%) |  | 139 (46.8) | 57 (52.8) | 82 (43.4) | 0.147 |  | 223 (43.0) | 84 (45.9) | 139 (41.4) | 0.354 |  | 0.306 |
| Calcified lesion, n (%) |  | 74 (24.9) | 32 (29.6) | 42 (22.2) | 0.165 |  | 116 (22.4) | 49 (26.8) | 67 (19.9) | 0.079 |  | 0.439 |
| Chronic total occlusion, n (%) |  | 27 (9.1) | 8 (7.4) | 19 (10.1) | 0.532 |  | 59 (11.4) | 17 (9.3) | 42 (12.5) | 0.312 |  | 0.344 |
| ACEi or ARB, n (%) |  | 242 (81.5) | 88 (81.5) | 154 (81.5) | 1.000 |  | 438 (84.4) | 149 (81.4) | 289 (86.0) | 0.205 |  | 0.285 |
| Beta-blocker, n (%) |  | 262 (88.2) | 92 (85.2) | 170 (89.9) | 0.262 |  | 468 (90.2) | 167 (91.3) | 301 (89.6) | 0.644 |  | 0.408 |
| SBP, mmHg |  | 129.8±24.9 | 131.8±27.3 | 128.7±23.4 | 0.298 |  | 144.3±25.0 | 146.0±24.5 | 143.3±25.3 | 0.251 |  | <0.001 |
| DBP, mmHg |  | 63.8±10.0 | 61.7±10.2 | 65.0±9.7 | 0.007 |  | 79.5±12.1 | 79.2±10.9 | 79.6±12.7 | 0.743 |  | <0.001 |
| Heart rate, beat/min |  | 72.3 ±12.0 | 72.4±12.5 | 72.3±11.8 | 0.911 |  | 75.5±14.3 | 75.7±14.0 | 75.3±14.4 | 0.790 |  | 0.001 |
| LVEDP, mmHg |  | 29.4±8.3 | 28.1±9.0 | 30.2±7.8 | 0.044 |  | 22.6±8.8 | 22.2±9.7 | 22.8±8.3 | 0.455 |  | <0.001 |
| CPP, mmHg |  | 34.4±6.9 | 33.6±6.2 | 34.8±7.2 | 0.143 |  | 56.8±11.1 | 57.0±10.7 | 56.8±11.4 | 0.816 |  | <0.001 |

ACEi, angiotensin-converting enzyme inhibitor; ARB, angiotensin receptor blocker; CKD, chronic kidney disease; CPP, coronary perfusion pressure; DBP, diastolic blood pressure; LVEDP, left ventricular end-diastolic pressure; LVEF, left ventricular ejection fraction; NYHA Fc, New York Heart Association functional class; SBP, systolic blood pressure
